# Continuity of care for TB patients at a South African hospital: A qualitative participatory study of the experiences of hospital staff

**DOI:** 10.1371/journal.pone.0222421

**Published:** 2019-09-18

**Authors:** Frederick Marais, Idriss Ibrahim Kallon, Lilian Diana Dudley

**Affiliations:** 1 Division of Health Systems and Public Health, Faculty of Medicine and Health Sciences, Stellenbosch University, Cape Town, South Africa; 2 Western Cape Government: Health, Cape Town, South Africa; 3 Division of Social and Behavioural Sciences, School of Public Health and Family Medicine, University of Cape Town, Cape Town, South Africa; Victoria University, AUSTRALIA

## Abstract

**Background:**

Ensuring effective clinical management and continuity of TB care across hospital and primary health-care services remains challenging in South Africa. The high burden of TB, coupled with numerous health system problems, influence the TB care delivered by hospital staff.

**Objective:**

To understand factors from the perspectives of hospital staff that influence the clinical management and discharge of TB patients, and to elicit recommendations to improve continuity of care for TB patients.

**Design:**

Participatory action research was used to engage hospital staff working with TB patients admitted to a central public hospital in the Western Cape province, South Africa. Data were collected through eight focus group discussions with nurses, junior doctors and ward administrators. Data analysis was done using Miles and Huberman’s framework to identify emerging patterns and to develop categories with themes and sub-themes. The participants influenced all phases of the research process to inform better practices in TB clinical management and discharge planning at the hospital.

**Results:**

The emerging themes and sub-themes were categorized into two overall sections: The clinical care management process and the discharge and referral process. Nurses expressed a fear of exposure to TB and MDR-TB due to challenges in clinical and infection-prevention control. Clinical hierarchies, poor interdisciplinary teamwork, limited task shifting and poor communication interfered with effective clinical and discharge processes. A high workload, staff shortages and inadequate skills resulted in insufficient information and health education for TB patients and their caregivers. Despite awareness of the patients’ socio-economic challenges, some aspects of care were not patient-centered, and caregivers were not included in discharge planning. Communication between the hospital and referral points was inefficient and poorly supported by information systems. Hospital staff recommended improved infection prevention and control practices and interdisciplinary teamwork in the hospital, that TB education for patients be integrated with hospital staff functions, with more patient-centered discharge planning, and improved communication across hospitals and primary health care levels.

**Conclusions:**

Interdisciplinary teamwork, more patient-centered care, and better communication within the hospital and with primary health-care services are needed for improved continuity of care for TB patients. Further studies on factors contributing to, and interventions to improve, continuity of TB care in similar hospital settings are needed.

## Introduction

South Africa has one of highest incidence rates of *Mycobacterium tuberculosis* (TB) infection and disease in the world [1], and is on the World Health Organization’s priority list of 30 high-burden countries [2]. With 18.9% of the adult population infected with HIV [3], an HIV prevalence in TB cases of 57% [1], and increasing drug-resistant TB (DR-TB) [4], large numbers of TB patients are hospitalized in South Africa [5]. With a national TB case detection rate of 64% [6], many “undetected” TB patients are among those admitted to acute hospitals with complex disease, or co-morbidities with HIV and other chronic diseases [5,7]. In addition to the high burden of TB and HIV, and fears of occupational exposure [8], hospital staff in South Africa encounter numerous health system challenges in highly “stressed” hospital working environments with serious resource constraints [9–11]. A complex range of national government policies and programs inform the delivery of public-sector health care within a historically fragmented health system. The National TB Programme (NTP) adopted the World Health Organization DOTS (Directly Observed Treatment, Short Course) strategy in 1995, and implemented it in primary health care (PHC) facilities and specialized TB hospitals, which provide long-term inpatient care for TB patients with complex disease such as MDR and XDR TB. General acute hospitals, despite admitting large numbers of active TB patients, were not included in the NTP [12]. Ensuring effective clinical management in acute hospitals and continuity of TB care between hospitals and PHC NTP services therefore remain challenging in South Africa [13–16].

A South African study of TB clinical management and referrals from an acute public hospital in the Gauteng province in 2003 reported that 50% (203/407) of patients attended PHC services following hospital discharge [13]. A similar study in KwaZulu-Natal in 2006 found that 29% of laboratory-confirmed TB patients reached a PHC clinic after hospital discharge, and 18% eventually completed treatment [14]. A descriptive study by the authors of this paper on care transitions in the Western Cape in 2011 found that approximately 36% (284/788) of hospitalized patients with TB reached PHC or specialized TB hospitals after discharge [15]. These studies, undertaken in different provinces several years apart, suggest that similar problems exist across the health system, with ongoing challenges in the clinical management and discharge of TB patients from acute hospitals. These descriptive studies, however, provide little understanding of the challenges experienced by health service providers in the clinical management and discharge of TB patients.

This qualitative study was therefore undertaken in collaboration with health services to understand factors influencing the inpatient clinical management and discharge of TB patients from the perspectives of staff employed in a public sector acute hospital managed by a Provincial Department of Health. Their experiences, concerns and recommendations were essential to identify contextually appropriate actions within the hospital, and across the hospital and PHC services, to strengthen continuity of care and improve TB patient outcomes. In this study, the concept of continuity of care refers to the provision of coordinated patient-centered care and services across different levels, specifically across hospital and PHC services, and disciplines over time [17]. The study supplements an earlier, descriptive study undertaken by the authors that used routine health information to assess continuity of care for TB patients between the hospital and PHC services in the Western Cape [15].

## Method

### Setting

This study was conducted in 2012 in a 1300-bed central academic hospital (CAH), the second largest public hospital in South Africa. The hospital received more than 50% of tertiary referrals from a catchment population of six million in the Western Cape Province [18]. The province had a high TB prevalence (681/100 000), and 8% of the adult population was infected with HIV [6].

### Design

We used a participatory action research approach throughout the research process to engage with hospital staff responsible for the inpatient care of TB patients [19]. Participatory action research is a form of self-reflective enquiry that engages researchers and participants to understand and improve upon their practices and the situations in which they operate [20]. This method therefore ensures the relevance of the research to the context, provides capacity building, and promotes the acceptability of the findings to support appropriate actions among participants to address the problems in a sustainable manner. Accordingly, the hospital staff influenced all phases of the research process, and their responses form an integral part of the production and translation of knowledge to improve practices in the clinical management and discharge planning of TB patients at the hospital.

As participatory action research is based on human relationships and trust, addressing disparities in the owning of spaces, power and influence between the researchers and the researched (study participants) was a key concern [21]. The researchers therefore maintained rigorous reflexivity on the processes and consequences of power constructions, as well as issues of trust between the researchers and participants [21,22]. Participant input guided the development and approval of the research design, questions and data collection tools. For example, senior clinicians and nurse managers recommended the inclusion of junior doctors in the study due to their key role in the clinical management and discharge of TB patients from the hospital. They also advised that ward administrators be included, as they are pivotal in coordinating the timely completion and submission of essential documentation, such as discharge letters and medicine prescriptions, by doctors and nurses. They further recommended that focus group discussions be used for data collection, rather than individual interviews, and that separate focus group discussions be conducted with nurses, ward administrators and junior doctors due to the prevailing hierarchy and perceived power differences that could impede participant contributions.

The study population therefore included student nurses (n = 4), professional nurses (n = 43), junior doctors (n = 6) and ward administrators (n = 7) employed in the hospital at the time of the study and who were involved in the clinical management and discharge of adult TB patients admitted to the hospital. Purposive sampling, in consultation with senior doctors and nurse managers, was used to recruit sixty staff in these categories from Internal Medicine and other clinical disciplines to participate in focus group discussions. Participants, initially informed about the study by senior doctors and nurse managers, were invited to participate through face-to-face and telephonic contact with the principal investigator. None declined to participate. The participants in the groups were compatible, as staff from the same clinical specialties, experience in similar clinical spaces and comparable interactions with TB patients were interviewed together [23]. Staff working in pediatric wards were excluded, as concurrent research was being conducted on the clinical management and discharge of hospitalized pediatric TB patients.

### Data collection

A semi-structured interview guide was developed and tested for contextual appropriateness, content relevance and ease of application with a representative sample of participants prior to data collection (S1 Guide). Eight focus group discussions of approximately one hour duration were facilitated by the principal investigator, assisted by a research assistant. Each focus group discussion included between six and 11 participants and was held during staff work break times in private rooms at the hospital, providing strict confidentiality. On the advice of senior nurse managers, the focus group discussions were conducted in English as the main common spoken language of hospital staff. Translators were available, but were not needed by the participants. The focus group discussions allowed participants to listen to each other’s statements, provide anecdotes and reflect on their experiences [23]. To maintain a bottom-up approach, the participants were reminded constantly about the importance of honestly communicating their lived realities and opinions during the group sessions. The interviews were audio-recorded with permission of the participants, using a Dictaphone, and supplementary written notes were taken by the researchers. Both the principal investigator and research assistant were engaged in data capturing to ensure data accuracy and reliability. They transcribed and cross-checked the coded scripts immediately after the focus group discussions to ensure full and accurate data capture.

### Trustworthiness

The researchers adhered to the importance of reflexivity throughout the data collection process [23], making personal experiences, opinions and preconceptions about the field of research explicit from the outset [24]. The principal investigator, a co-investigator in the main study [15], was a participatory and health systems researcher with a PhD in public health from a nursing background, and was employed as a senior lecturer in community health. The research assistant was a Master’s-level public health researcher with expertise in health systems. Neither were involved in clinical TB care and discharge planning, but they were familiar with the context of care provision in the CAH. The principal investigator and the third author had established a relationship with the hospital through a descriptive study [15] and, as joint employees of a university and provincial health department, were mandated to support the health services with relevant health systems and public health research.

Trustworthiness was enhanced further by closely observing the self-reflexivity of the researchers as they came into contact with the participants [19,21,25]. The themes used for the focus group discussions were identified through a participatory process. The structure and facilitation of the focus groups provided an environment that was conducive for the participants to express their perceptions and experiences honestly. The researchers clarified the responses and verified the contextual appropriateness of the data coding and emerging themes with the participants. In addition, the two primary researchers and the third author, an expert in TB management and health systems research, verified the thematic analysis and discussed disagreements to reach consensus. The key findings were also discussed with and verified by senior nurses who formed part of the study sample.

### Data analysis

We used the Miles and Huberman framework for qualitative data analysis, applying steps for data reduction, data display, and drawing and verifying conclusions [26]. These involved memo-ing (close reading of the whole manuscript) to identify and manually pre-code key emerging patterns linked to the final themes, followed by electronic coding with the assistance of the QSR International NVivo 10 software package. Ensuring inter-coder reliability (for which the researchers coded the same data and compared codes and themes) enabled the researchers to verify and recode the data where necessary, and made it possible to develop categories with themes and sub-themes (S1 Analysis).

The final categories, key themes and sub-themes are presented with quotes from the participants to illustrate and add depth to the findings. Quotations are used verbatim and are presented in italics, followed by a unique number indicated in brackets (for example #1), to provide context for the data.

### Reporting

The COREq framework, a 32-item checklist for qualitative research, was used to ensure the critical reporting of important aspects of the scientific design and findings of this qualitative participatory study (S1 Checklist) [27].

### Ethics

Ethical approval for the study was obtained from the Health Research Ethics Committee of Stellenbosch University (Ref: N09/05/149), the hospital and the Western Cape Government: Health. Written consent was obtained from all the participants, who were informed of the voluntary terms of involvement in the study and the use of audio-recordings, and were assured of the confidentiality and anonymity of the information they shared. The interview transcripts were coded and personal identifying details were not collected. The research process did not interfere with health-care service delivery. Participants were not paid for their participation in the study. As a gesture of appreciation, refreshments were served after the focus group discussions.

## Results

The study sample comprised 60 hospital staff (student nurses, N = 4; professional nurses, N = 43; ward administrators, N = 7; junior doctors, N = 6). The female (n = 56) and male (n = 4) participants represented diverse ethnicities and were all conversant in English. The predominance of females and nurses was representative of the workforce in the hospital. Nurse participants in the focus group discussions were mostly from internal medicine (n = 18), surgery (n = 14), emergency services (n = 8), and obstetrics and gynecology (n = 7), as presented in Table 1. Data saturation was reached within the sample.

**Table 1:** Participant numbers and characteristics (medical module and occupation).

Participant no.	Clinical specialty	Occupation
1–8	Emergency services	Professional nurses (n = 8)
9–19	Internal medicine 1	Professional nurses (n = 9) Student nurses (n = 2)
20–26	Obstetrics and gynecology	Professional nurses (n = 7)
27–33	Internal medicine 2	Professional nurses (n = 5) Student nurses (n = 2)
34–40	Surgery 1	Professional nurses (n = 7)
41–47	Surgery 2	Professional nurses (n = 7)
48–53	Internal medicine 3	Junior doctors (n = 6)
54–60	All	Ward administrators (n = 7)

Three final categories emerged from the data analysis, specifically the clinical care management process, the discharge and referral process, and recommendations for health systems change. These, along with their key themes and sub-themes, are illustrated in Table 2 and described below.

**Table 2:** Categories, key themes and sub-themes from participant responses.

Categories	Key themes	Sub-themes
**Clinical care management process**	Fear of exposure to TB infection	MDR-TB (multidrug-resistant TB)
		Clinical challenges
		Patient health-seeking behavior
	Task shifting and not sharing	Insufficient time and staff shortage
		Lack of health education resources
	Poor documentation and coordination	
	Hierarchy and territory	
**Discharge and referral process**	Poor discharge planning	Lack of patient-centeredness and preparedness
		Insufficient collaboration between hospital and referral points
	Socio-economic conditions of the patients	Patients’ living conditions
		Health literacy and personal agency
**Participant recommendations for health system actions**	Strengthen TB-IPC	
	Improve communication at and across hospitals and PHC levels	
	Improve TB education for patients and staff	
	Patient-centered discharge planning	

### Category 1: Clinical care management process

The participants exposed four key influences (themes), each with its own interconnected factors (sub-themes), which affected the quality of the hospital’s TB clinical management process. These were fear of exposure to TB infection, the shifting and not sharing of patient care tasks, poor standards of documentation and coordination of patient care, and traditional hierarchy and territorial roles amongst hospital staff.

### Theme 1.1 Fear of exposure to TB infection

#### Sub-theme 1.1.1 MDR-TB

Many of the nurses expressed a fear of exposure to TB, which was related to work practices at the hospital. They were concerned about the occupational and environmental exposure of patients and staff to TB, especially MDR-TB, as a result of the standard of operational and clinical management of MDR-TB patients as stated by a professional nurse.

*Especially with MDR where there is a lot of exposure to MDR patients*. *When they come in and you don’t know*, *afterwards you find out or if they are too sick to tell* (#6, professional nurse).

Another nurse explained that some of the TB patients were well known, and thus their suspicion and fear increased:

*So you might admit someone that’s said to you rely on the doctor to do the screening*. *But you already know this person has been here before he/she has not been in an open ward because they have missed treatment*. *It could be MDR-TB*, *the risk is high*. *But it seems you don’t have that information to make a clinical judgment about*, *is that right*? (#9, professional nurse).

A junior doctor also echoed strong concerns about unknown MDR-TB patients placed in general wards:

*We can suspect an MDR type of situation as well*. *We usually find that out ourselves*. *And that takes a bit of time when the person is sitting in a normal ward whatever the case is*, *and potentially has defaulted treatment* (#48, junior doctor).

#### Sub-theme 1.1.2 Clinical challenges

The majority of participants raised concerns about unclear or incomplete patient records, a lack of sufficient beds and respiratory-isolation facilities, a low index of TB suspicion among hospital staff, and poor role modelling by senior hospital clinicians. Several nurses mentioned the confusion and skepticism created by senior staff who do not wear the required personal protective equipment, such as face masks, and their reluctance to confront them about their incorrect practices, as reflected in a professional nurses comment.

*And so you don’t wear the mask and then you are exposed to those* [TB] *patients*. *Only afterwards it’s like*, *‘No this patient is* [TB] *positive*!*’*, *and then we make a plan to put the patient in a single room*. *There is a lack of negative pressure ventilation*, *so we are always exposed* (#6, professional nurse)

One of the participants reported alarm about the fact that patients in shared wards awaiting TB treatment posed a high risk of cross-infection to other patients and hospital staff.

*Clinical picture*? *When they* [TB patients] *come it’s normally full by us so they will lie in the passage waiting and we are exposed to them and in their active* [TB] *phase* (#9, professional nurse).

Ensuring patient adherence to respiratory precautions during hospitalization was another clinical challenge of concern.

*I think there is a real problem with keeping people in isolation because they want to come out*. *It is a major problem because of the risk to us and other patients* (#9, professional nurse).

#### Sub-theme 1.1.3 Patient health-seeking behavior

Concealment by patients of their TB diagnosis and treatment on admission to hospital was mentioned often by the participants. They felt that such a lack of disclosure contributed to breaks in the continuity of TB treatment and impeded effective infection prevention and control (IPC) measures in hospital.

*They* [patients] *are not honest … They keep their medication in the locker and don’t tell us … They will keep their own medicine and keep taking it*. *Then only when they run out they will say*, *then you might learn that the person has pulmonary TB which is three weeks or two days down the line* (#6, professional nurse).

### Theme 1.2 Task shifting and not sharing

Most participants indicated that nurses and junior doctors lacked clarity concerning their roles, responsibilities and requirements in terms of TB continuity of care. There was no standard approach for TB “induction” (information and education) of newly diagnosed patients, resulting in a culture of blame amongst the health-care workers. A nurse explained that:

*The general impression is when the patient leaves the ward it’s no longer the ward’s responsibility … It is the transit lounge … It is the manager’s responsibility … So*, *whatever happens down there they handle that* (#10, professional nurse).

Another felt that:

*When the patient comes to the ward we must be the primary people giving the information*, *health information*. *Sometimes they get diagnosed and then within that first week*, *less than the 12 days*, *they get discharged*. *So in that time they might be so sick you cannot get through with education*. *So*, *there’s also a lack there* (#11, student nurse).

The student nurses reported that, on the instructions of ward management, they were not allowed to offer any TB-related health education to patients and their families. Most of the professional nurses were of the opinion that the doctors should offer TB information and education to the patients, as they were accountable for their medical care. In contrast, most of the junior doctors felt that the nursing staff were best positioned to offer health education to TB patients, as they spent more time with the patients. A nurse and a junior doctor argued that the PHC services should be responsible for patient education:

*At the primary health care level*, *they are supposed to tell them* [TB patients]. *Because the primary health care level they are supposed to tell them they must prevent being in the crowd*, *and when they are in the house they must prevent being amongst the other siblings* (#7, professional nurse).

A junior doctor stated that:

*The clinic is the first place of care the patient goes to*, *so if people working there is unable to give advice*, *education*, *and health care to the patient*, *then the patient will default*, *like into a tertiary hospital* (#51, junior doctor).

#### Sub-theme 1.2.1 Insufficient time and staff shortages

The nurses and junior doctors repeatedly expressed concerns about insufficient time for structured and comprehensive TB education for patients due to the overwhelming workload. Additional concerns raised were inadequate numbers of staff and a lack of TB expertise amongst staff. A nurse explained that:

*The only thing that we do is that we sometimes inform the people that accompany the patient*. *Sometimes there is time for us to give that information and we also have the information on the walls just to say what do you expect*, *what do you see when you have TB*. *That’s what we can do from our side* (#1, professional nurse).

A junior doctor added that:

*I also feel that quite a lot of patients get diagnosed with TB*. *They come with weird presentations and at the end of the day it comes down to TB*. *So we start with empirical treatment*, *we are supposed to notify and also educating them*, *it’s a very brief process*. *Unfortunately*, *there is not as much time as we would like to have* (#50, junior doctor).

Another junior doctor agreed that:

*What compromises the care of TB patients is time*, *absolutely time*. *We just don’t have time to do all of the stuff that’s necessary to look into their background whilst as we would like to do that* (#52, junior doctor).

A nurse stated:

*You know*, *it’s not really making the time*, *for the patient to understand how important it is to drink his tablets and really spend time*. *They don’t do it*. *Everybody is in a rush*. *I need the bed you must go you’ve got TB*, *your lungs are affected*. *Finished* (#5, professional nurse).

#### Sub-theme 1.2.2 Lack of health education resources

All participants highlighted the lack of health education resources, such as printed materials and videos, in different languages. Several participants expressed the need for appropriate resources to be made available to patients during their hospitalization and to take home on discharge. Participants spoke of the reality of language and health literacy barriers, and the need for timely and repeated patient education.

*I think it’s very important for constantly educating*. *It depends on the stage of their mental side you know*. *So*, *you will actually give some education but a week or two weeks later the mental status is better*, *and then you realize that the patient can get much more information in*, *so he needs some more education* (#12, professional nurse).

A junior doctor added that:

*It’s always a language barrier*. *Quiet often we rely* [for translation] *on nursing staff*, *often it’s the cleaner*, *often it’s the family member or any other patient and you never really know what they tell you*! *I actually think there is a big*, *big gap*! *And it all starts here at the end of the day* (#49, junior doctor).

### Theme 1.3 Poor documentation and coordination

According to the participants, inadequate communication between hospital staff resulted in poorly coordinated patient care. The absence of clear documentation of TB cases (suspected/confirmed), partial handovers on patient admission and transfer, poor notification of TB cases, and the absence of an electronic hospital database of TB patients were highlighted frequently.

*In terms of the instructions*, *if it says go two days to* (a local) *clinic for example*, *is that done in different languages*? *I think it’s the doctor who should actually explain to the patient as to why he needs to be in isolation in the ward because of the other patients and the nurses*. *I don’t think the nurses should do that because they listen more to the doctor than to the nurses*. *And I mean*, *it’s the doctor that do the diagnosis in the first place* (#13, professional nurse).

Lack of and inaccurate records concerning the TB status of patients were major concerns for the nurses and ward administrators. A nurse explained that:

*… the concern is when we admit the patient sometimes*, *we don’t know if they have pulmonary TB*. *We’ve reviewed the medical notes for that ward and the documentation is really not existing* (#14, professional nurse).

A ward administrator stated that:

*They* [doctors] *do not do it* [proper coding of patient records], *you can tell them several times*. *They have a certain time of the year that they rotate*, *they work in internal medicine and then they go to obs and gynae*. *With every new group that comes*, *you will explain to them*, *doctor please fill in the ICD code* [International Classification of Disease for TB]. *They will do it for the first time after that it won’t happen* (#54, ward administrator).

Another ward administrator stressed the responsibility of doctors for ICD-10 coding:

*It’s the doctor’s responsibility*, *you won’t even know whether that code is right or wrong*. *I mean if the doctor writes the code down there who are you to come and say that is the wrong code*? (#55, ward administrator).

### Theme 1.4 Hierarchy and territory

The nurses felt that professional barriers between themselves and the doctors prevented effective transdisciplinary communication and practice and impacted negatively on the continuity of TB care. Most were reluctant to track patients’ electronic TB results and had an aversion to consult and confront doctors (the “other”) as reflected in a nurse’s comment.

*Sometimes the patients are in for a couple of weeks in the ward*. *With all the tests that the doctor is doing and then after four weeks then the doctor obviously tells us that the patient got TB or they detect TB spine by biopsy or what* (#4, professional nurse).

One of the nurses took personal initiative to track outstanding clinical results:

… *sometimes as a nurse you will see that the patient is very sick*, *and he looks like somebody with TB or whatever*. *And then you will probably go to the computer just to check is nothing out on this man you know*? *We can’t treat this person if something* [results] *is not up there*. *But then you go and you check on the computer to see if the sputum result is out or not* (#2, professional nurse).

### Category 2: Discharge and referral process

The participants highlighted poor discharge planning and the socio-economic conditions of TB patients as two major aspects that affect the quality of the TB discharge and referral process between the hospital and PHC services. In turn, these influences affect the continuity of TB care.

### Theme 2.1 Poor discharge planning

#### Sub-theme 2.1.1 Lack of patient-centeredness and preparedness

The nurses felt that a patient-centered approach was important in preparing TB patients for discharge, but was lacking in the hospital TB discharge-planning process. They felt that an assessment of the socio-economic conditions of the TB patient was generally inadequate; the documentation of patients’ personal contact details was poor; and there was scant patient, family and/or caregiver involvement in the discharge planning. Linguistic requirements and the health literacy of patients were not adequately considered; and there were gaps in discharge information, including outstanding clinical results, and in treatment and referral instructions offered to TB patients and their caregivers.

*We get the discharge letter from the doctor and we’ll say ‘ok the results are outstanding’*. *Then we take that letter and they* [the patients] *must take it to the Day Hospital*, *and say on the next day they must now sort that out*. *I don’t know from there on if they* [PHC] *look up results*, *what they do I don’t know* (#6, professional nurse).

Another nurse explained:

*Sometimes we do send the patients down* [to Pharmacy] *because the doctor is in a hurry to get the bed for another patient*. *Then we don’t see the patient*, *we don’t know if they had picked the tablets up* (#7, professional nurse).

A ward administrator suggested that:

*Ok*, *just to double check*. *I think it will be safer if we can get the information* [contact details] *from the patient himself because there might be a lot of mistakes*, *sometimes there are old addresses and people don’t say and we sit with the old address*. *So for the accuracy*, *better to talk to the patient (*#54, ward administrator).

One of the junior doctors expressed the need for a designated hospital TB discharge coordinator to strengthen discharge planning:

*They need to see every single patient who is diagnosed with TB and who gets notified before the patient leaves*. *And get somebody who can speak to them and give them information and stay in contact with those people … And also to coordinate the whole notification*, *TB referral and clinical referral letters*. *Someone needs to stay on top of that*, *somewhere between us and the ward administrator*, *we are missing something and the patient* (#49, junior doctor).

#### Sub-theme 2.1.2 Insufficient collaboration between hospital and referral points

The findings indicate that PHC services were not part of the discharge-planning process, resulting in discharge letters containing unclear or incomplete referral information. A ward administrator reported that:

*Sometimes there is a case where one of the nurses of the* [PHC] *clinic will call because they received the forms* [patient referral] *with inadequate information on it*, *then we’ll fax it through* (#56, ward administrator).

A junior doctor suggested potential for electronic communication to improve patient referrals across health services:

*Won’t it be cheaper and more effective if the hospital sends emails or any form of communication to the clinic* [PHC]? *Instead of the patient going with the letter*, *which the patient is not going to take to the day hospital* [PHC] (#48, junior doctor).

There was also no follow-up with or feedback on patients accessing PHC services following discharge, nor any communication from PHC services when referring TB patients to the hospital. A nurse explained that:

*We have a problem*. *We would like to know when the patient started on TB treatment*, *and where*. *When they have been referred you just know they are on TB treatment* (#6, professional nurse).

One of the junior doctors stated:

*I believe that the hospital should have a good communication network*, *a working network with all the primary care clinics to reduce the incidents of the patients coming to the hospital*. *Because that doesn’t have to happen*, *that we have such a high loads of TB patients who come here*, *and HIV-positive patients* (#49, junior doctor).

### Theme 2.2 Socio-economic conditions of patients

#### Sub-theme 2.2.1 Patients’ living conditions

The participants stated that most patients diagnosed with TB were from impoverished communities in the Western Cape. Extreme poverty, conflicting survival priorities, lack of family support and ongoing TB exposure (returning to the “pool of infection”) were highlighted repeatedly. They saw a relationship between a lack of home and community support for TB patients and poor continuity of care following discharge from hospital as reflected in a nurse’s comment.

*There’s nobody to help them* [TB patients]. *Those are the patients that always come back very sickly to your department*. *Now you help them*. *They are better*, *they go home they fall back*, *they default and come back*. *Because not everybody cares* (#27, professional nurse).

The contrast in care for patients in hospital and at home following discharge was another concern.

*Especially for the patient who comes here* [hospital] *and say ‘ok I’m going to live in peace here now’*. *Especially when you look at the patient’s circumstances at home*, *he comes here at least ‘somebody’s gonna wash me*, *I’m gonna to be fed here*. *I’m going to relax*. *I’m going to get better’ and all of a sudden* [we say] *‘No*, *you are going home now*! (#28, student nurse).

The challenging living conditions of some patients also had a negative impact on their opportunities for respite care at specialized TB hospitals.

*If those places* [specialized TB hospitals] *don’t take the patient*, *then we do have a real problem*. *For patients to get in*, *they must have a next of kin*, *they must be able to sign consent to say what’s going to happen to their bodies*, *either research or cremation or whatever*. *But then they must also have somebody who takes responsibility for them*. *And most of the patients don’t have that* (#29, professional nurse).

A connection between socio-economic conditions, continuity of TB care and the increased risk for drug-resistant TB was indicated by most of the participants. A nurse stated that:

*I think the problem is outside*. *The patient leaves here*, *the patient don’t usually go for the tablets and that’s why the patient gets MDR or XDR*. *There are problems outside like someone doesn’t have the money to go the clinic*, *economic reasons everything*. *So there is variety of problems that one deals with*. *We can’t just write it off in the hospital because we don’t know what happened when the patient leaves this place* (#37, professional nurse).

#### Sub-theme 2.2.2 Health literacy and personal agency

The nurses suggested that patients’ low levels of education, poor understanding of health and health-seeking behavior, and attitudes as submissive recipients of care, contributed to the failure in continuity of TB care.

W*e have a big problem with patients absconding from the hospital*. *… if they hear the word discharge*, *they want to leave the hospital as soon as possible but most of them are with complications and then they abscond without treatment*, *without consent*, *some of them are TB patients* … (#23, professional nurse).

Several nurses stressed that patient health-seeking behavior and inefficient hospital systems contributed to the breakdown in continuity of TB care.

*The problem is this*, *we’ve got the patient*, *when they hear the word discharge*, *they phone their people and say* [to the nurses] ‘*look I’m going home now’*. *They will give you all the promises to fetch their medication and all that*. *Now the medication comes* [from pharmacy to the ward], *we sit with that medication and it takes them* [patients] *up to three days to come back again*, *or simply don’t arrive*. *Then we have to send those medications back*. *That is our system* (#26, professional nurse).

A junior doctor also identified barriers to continuity of TB care created by factors related to the health system and patient behavior:

*Discharged patients get TB medication for five days*. *Now you are telling me somebody can get discharged on a Thursday and ideally they need to be at the hospital again like Monday because now they need to get medication for the next month*? *They just don’t*! [collect their medication] (#50, junior doctor).

### Category 3: Participant recommendations for health system actions

The proposed actions to improve continuity of care for TB patients were grouped around TB IPC measures, health services communication and coordination, TB education and patient-centered discharge planning.

### Theme 3.1 Strengthen hospital TB infection prevention and control (IPC)

The need for improved hospital IPC practices was stated repeatedly by the nurses and junior doctors, who recommended: (a) role modelling with accountability, with particular reference to senior doctors, nurses and managers, (b) designated respiratory-isolation facilities and wards with adequate negative pressure rooms managed by expert staff, (c) a rapid TB notification and contact screening system with access to electronic records for health-care workers, and (d) a system that enforces and sustains a high index of suspicion (“TB in all”) amongst all hospital staff.

### Theme 3.2 Improved communication and coordination at and across hospital and PHC levels

To improve communication and coordination between the hospital and PHC services, several participants recommended: (a) clarifying roles, responsibilities and accountabilities, (b) enhancing the quality of verbal communication and written documentation, and (c) providing standardized TB documentation in the hospital to promote continuity of care. They proposed that the latter include a TB discharge checklist and an electronic TB patient register that is accessible to hospital and PHC-based health-care workers. They also felt that early discharge notifications to and engagement of staff at the PHC referral point were needed.

### Theme 3.3 Improved TB education for patients and staff

Most of the participants stressed the need for improved patient education about TB and continuity of care. They identified several critical components: (a) delivery of timely TB education in the hospital and PHC settings, (b) ensuring that the patient receives adequate information about continuing and completing TB treatment, (c) confirming that the patient understands the treatment requirements prior to discharge and on accessing the PHC services, and (d) supplying supportive TB information resources to any family members and caregivers of the patient. Some nurses and junior doctors argued that the above actions should be integrated into nursing care. The provision of in-service training on broader aspects of TB care for multidisciplinary hospital staff (day, night and agency workers) was another critical recommendation.

### Theme 3.4 Patient-centered discharge planning

The recommendations made by many participants’ for an improved patient discharge-planning process focused on the “person”, including: (a) early assessment of the socio-economic conditions (barriers and enablers) of TB patients, (b) identification of and addressing patients’ language and health literacy requirements, (c) early engagement with the patient and family/caregivers to better promote patient agency, and (d) provision of a “discharge pack” comprising health education resources, treatment and IPC instructions, contact numbers of local PHC services, together with a discharge letter in English and the main spoken language of the patient. A further important recommendation was the availability of a hospital-based TB discharge coordinator or counsellor to manage patient-centered continuity of TB care at and across the hospital and PHC settings. The consensus was that such a role would address the limited time available for patient education and health promotion due to the pressures of clinical responsibilities and workload experienced by nurses and junior doctors.

## Discussion

The participants’ insights revealed several challenges and proposed actions to improve continuity of care for TB patients discharged from an acute hospital. We discuss these in two categories, namely clinical care management processes and discharge and referral processes. The participants’ recommendations for health systems actions are integrated into the discussion of these categories.

The participants’ responses on clinical care management processes revealed extensive fear of exposure to TB infection, particularly MDR, which was attributed to poor hospital IPC systems and insufficient availability and use of patient-isolation facilities. Similar fears of infection and IPC system failures were identified in previous studies at the CAH, and as the main occupational concerns of hospital nurses in the Western Cape and in hospitals in the Free State province of South Africa [8,16,28]. Occupational TB is a significant risk for health workers in low- and middle-income countries (LMICs) [29], including South Africa [30,31], where the annual incidence of infection among health-care workers is substantially higher (>20%) than that (7.2%) in other high-incidence countries [32].

Some of the participants proposed that the hospital should establish clearer accountability for IPC and improve compliance with IPC practices, such as designated respiratory-isolation facilities, rapid TB notification, and screening of household and hospital contacts. Although IPC is one of six national quality of health-care priorities in South Africa, only 50% of health facilities comply with the national standards for IPC [33]. Hospital IPC for TB therefore warrants further attention to protect patients and health workers, and to enable health workers to provide the necessary care for TB patients without fear of infection.

The lack of guidelines and unclear roles and responsibility with regard to patient education, clinical management and discharge planning within the hospital were identified as important factors contributing to poor continuity of care for TB patients. Both nurses and junior doctors expressed the view that it was the other discipline’s role, or that of PHC services, to provide TB-related information and education for patients. They also expressed the view that disciplinary silos and hierarchies created barriers to interdisciplinary communication and practice, with a negative effect on the clinical management and continuity of care for TB patients.

These shortcomings were echoed in studies at the CAH, which found an absence of standardized approaches to inter-professional teams, limited perceptions of teamwork amongst staff, and substantial barriers to inter-professional teamwork, which had an impact on patient care [16,34]. Participants felt that communication about and coordination of the clinical management and discharge of TB patients could be improved by clarifying roles and responsibilities, and by improving communication and inter-professional teamwork. Numerous interventions have been developed to improve inter-professional teamwork in delivering patient-centered care, with evidence that inter-professional education may improve adherence to clinical guidelines, clinical processes and patient outcomes [35]. Interdisciplinary collaboration is also regarded as a core competence for health professionals to respond more effectively to challenges in health systems [36].

Communication with TB patients about their illness was limited by the high workload, staff shortages, and time constraints of busy ward staff. The pressure to empty beds also resulted in TB patients leaving hospital without adequate counselling. The absence of appropriate health-promotion materials in the patients’ home languages, and the low literacy levels of TB patients, were further obstacles to providing health education. This was compounded by the health professionals’ own lack of knowledge and skills to provide counselling, and a lack of health education resources in different languages for staff and patients in the hospital.

Several nurses wanted more time for patient education and counselling, appropriate TB information and education resources, and training on TB care as part of a multidisciplinary team. There is evidence that patient education and counselling improve treatment adherence [37], but few studies have assessed the effects of counselling inpatients on continuity of care between hospitals and other levels, nor the roles of different health professionals in providing such counselling and education for TB patients.

Poor communication between members of the hospital staff was experienced through inadequate documentation of TB in clinical records, particularly by clinicians, partial information provided in handovers or internal patient transfers, and the absence of an integrated hospital TB information system. The delayed or incomplete knowledge about patients’ TB diagnosis further limited clinical decision-making on and communication with TB patients. These findings echo the results of a national audit of health facilities, which found that communication in health facilities was poor (64% compliance), and only 46% of facilities used health technology appropriately [33]. The descriptive study at the CAH also found poor integration of information systems within the hospital, and between the hospital and other levels of care, to support continuity of care for TB patients [15]. A recent study at the CAH also revealed inaccurate ICD coding, which limited the quality and completeness of data being communicated to other levels of care [38].

The participants in this study wanted better verbal and written communication, and suggested that the standardized National TB Control Programme (NTP) guidelines and documentation be adopted in the hospital to facilitate links with NTP services. They also recommended adopting electronic information systems for recording, notifying and sharing information within the hospital and with PHC services. Links between acute hospitals and the NTP had been improved in other high-TB burden countries by adopting the NTP tools, including electronic recording and reporting systems, in the clinical management of TB patients [39,40]. This contributed to improved case finding, quality and continuity of care for TB patients in India, China and Indonesia [39].

Most of the nurses expressed concerns about insufficient patient-centeredness in the discharge planning of TB patients. They felt that patients’ socio-economic conditions, the lack of community resources, and the need for interdisciplinary care were not adequately considered in the discharge process. The patient and his or her family were also not included in the discharge planning. Along with the lack of patient education and counselling, this undermined the patients’ role and agency in their own health care. The participants suggested including an assessment of the patients’ socio-economic conditions as part of the discharge process, and felt that earlier engagement with the patient and family on these issues was necessary.

Although patient-centered care is prioritized in the national and provincial health plans in South Africa [41,42], a national audit of health facilities found that only 30% of facilities met the criteria for “positive and caring attitudes” [33]. Socio-economic factors are important in the prevention of TB [43,44], adherence by TB patients to treatment [45], and in the discharge-management process [46]. In the Western Cape, formal housing and employment were found to decrease the risk of default from TB treatment, and alcohol and drug use increased the risks of default in MDR TB patients discharged from specialized TB hospitals [46].

Despite the awareness of the importance of social context in a patient’s health-care management, there is little evidence of interventions to integrate this aspect into clinical management and discharge planning. A scoping review found some “expert-based” frameworks for including the social context of patients in discharge planning in high-income settings. However, there was little integration of evidence from the literature, and inadequate field testing of these frameworks [47].

The lack of involvement of PHC services in the discharge planning of TB patients, and inefficient communication–including incomplete information in patient discharge letters and the absence of electronic information links between the hospital and PHC services for referrals and follow-up of TB patients–were perceived as major barriers to continuity of care. Similar findings in other high-TB burden countries of poor links between hospitals and PHC TB services influenced strategies to include general hospitals in the NTP, including coordinating structures across levels and sectors to facilitate better communication and information sharing [39,48,49]. Interventions to improve the coordination of care between hospitals and other levels of care, such as discharge planning [50], care pathways [51] and interactive communication between specialists and primary care providers [52], have also been shown to reduce the duration of hospital stay and readmissions, and to improve patient outcomes for a range of chronic diseases.

This study has identified important factors contributing to poor continuity of TB care from the perspective of hospital staff, who also provided recommendations for change that are aligned with global and national priorities and interventions for improving continuity of TB care between hospitals and the NTP. These findings contributed to an intervention to improve the continuity of care for TB patients between the CAH and PHC services in the Western Cape. Several of the participants’ recommendations for action to be taken in health systems were thus implemented at the CAH, including enhanced hospital-based TB IPC processes, systems and tools to improve discharge planning, staff training and support for patient counselling and education, and electronic information systems to support communication and coordination within the hospital and across the hospital and PHC services.

### Limitations and strengths

There was extensive participation in the study by a large sample of nurses across a range of seniority and clinical areas in the hospital, but smaller numbers of junior doctors and ward administrators participated. Senior doctors were approached to participate, but they and the nurse managers recommended that the study focus on the perspectives of junior doctors, who dealt more directly with the discharge processes of TB patients than senior doctors. Further attempts were made to sample senior doctors, but their extensive clinical and managerial responsibilities made them inaccessible at the time of data collection. The participants’ perspectives, therefore, largely reflect those of hospital nurses. The restricted input of other health professionals is a limitation in a hospital environment, where interdisciplinary teams are an important component of the problem at hand and potential solutions. The study did not include health workers in PHC settings, or TB patients and the community, and therefore may not have identified key barriers to the continuity of TB care from a PHC, patient or community perspective.

The study was conducted by a small group of researchers, who also had academic and service responsibilities and limited resources for the research. The team conducted multiple studies to inform, implement and evaluate interventions at the CAH over this time period so as to improve continuity of care for TB patients. The limited capacity, along with the prioritization of local feedback and action, resulted in delays in publication. A mixed-methods evaluation of the intervention at CAH, which drew on this study, found significant improvements in continuity of TB care, although several elements of the clinical care management processes, and the discharge and referral processes within the hospital, needed further support. In particular, challenges in IPC, interdisciplinary teamwork, patient-centered care, and communication and coordination within the hospital persisted. The findings of this PAR therefore remain relevant in this and similar acute hospital contexts in South Africa in the absence of national policy or programs to improve continuity of care for TB patients discharged from hospital.

### Implications for policy and practice

Many ‘undetected’ TB patients in South Africa may be in acute hospitals, where continuity of care and TB outcomes are poor [13–15]. To improve case detection, patient outcomes and performance, the NTP should strengthen linkages between acute hospitals and NTP services. This study provides important insights from hospital staff on barriers to and facilitators of continuity of TB care, and recommendations for action. More attention needs to be paid to improving patient-centered TB care, interdisciplinary teamwork, and coordination and communication between hospitals and PHC services in relation to health policies and programs in South Africa. Lessons can be learnt from other chronic disease programs, particularly HIV care, where a strong patients’-rights paradigm has resulted in a more patient-centered approach [53].

### How do the findings inform further research?

Further studies are needed in similar, acute hospital settings in South Africa and Sub-Saharan Africa to understand the challenges to improving the delivery of patient-centered TB care, interdisciplinary teamwork, and communication and coordination within hospitals and with PHC services to support continuity of TB care. Studies of the perspectives of a broader group of hospital staff, PHC staff, TB patients and community members are also necessary for a comprehensive understanding of the enablers of and barriers and opportunities to improve TB continuity of care between hospitals and PHC services. High TB-HIV co-infection rates, and co-morbidities of chronic non-communicable diseases in TB patients [54,55], suggest that research on the continuity of TB care should draw on evidence on continuity of care for HIV and chronic disease patients. It also suggests that more research should be conducted from a patient and systems perspective to address barriers to and facilitators of continuity of care across diseases.

## Conclusion

Hospital staff highlighted several factors in the clinical management and discharge of TB patients that undermined the continuity of TB care. In particular, challenges were identified and recommendations were made to enhance TB IPC systems and practices, interdisciplinary teamwork, patient-centered care, discharge planning, and communication within the hospital and with PHC services. Contextual challenges included a high workload, staff shortages, and the poor socio-economic circumstances of TB patients. In accordance with the principles of participatory action research, several of the participant recommendations for actions to address the problems were implemented in an intervention package developed with hospital and PHC staff. Further studies to understand and inform interventions to improve the continuity of TB care in similar hospitals in South Africa and other settings are needed. Importantly, TB patients and their families, together with hospital and PHC staff, should be engaged in such research to ensure contextually appropriate interventions.

The authors declare that they have no competing interests.

## Supporting information

S1 Guide Focus group semi-structured interview guide. (PDF)

S1 Analysis Coding tree. (PDF)

S1 Checklist COREQ (COnsolidated criteria for REporting of Qualitative research). (PDF)
