# Supplementary material for: Continuity of care for TB patients at a South African hospital: A qualitative participatory study of the experiences of hospital staff
Source: PLoS One. 2019 Sep 18;14(9):e0222421. doi: 10.1371/journal.pone.0222421 (PMC6750596; doi:10.1371/journal.pone.0222421)
Supplement: S1 Guide — (PDF) [file pone.0222421.s001.pdf]

S1 Guide: Focus Group Semi-Structured Interview Guide

Care Management Process

1. What has been your experiences of providing care for people with TB and people co-infected with TB and HIV?
2. What steps are taken when someone gets admitted to hospital?
3. How do you become aware of the TB and/or HIV status of a patient on admission?
4. How do patients, their families and carers, receive health promoting information about TB?
5. What approach is used to manage any language barriers?
6. What is the standard of the patient documentation that you receive?
7. How do you ensure that the clinical management is effective?
8. Some people have TB or TB/HIV how is that care managed across all these disciplines?
9. Explain the working relationship between Nurses, Doctors and Ward Administrators.
10. Are there any other additional factors that affect the management of TB patients?

PHC Referral Process

11. What steps are taken to plan the discharge of a patient from hospital?
12. What steps are taken to refer a patient from the hospital to a local PHC clinic?
13. What process is followed to communicate outstanding results, such as TB or HIV, to the referral point?
14. Are there any other additional factors that affect referrals to PHC services?

Suggested Recommendations

15. What measures or strategies do you have to maintain a high index of suspicion that somebody might have TB?
16. What would make the care and management of patients at the hospital better?
17. What would make the discharge of patients work better?

18. Anything you feel we have overlooked, thinking about TB management, TB/HIV, discharge process that will help make your work easier?
