# Supplementary material for: Continuity of care for TB patients at a South African hospital: A qualitative participatory study of the experiences of hospital staff
Source: PLoS One. 2019 Sep 18;14(9):e0222421. doi: 10.1371/journal.pone.0222421 (PMC6750596; doi:10.1371/journal.pone.0222421)
Supplement: S1 Analysis — (PDF) [file pone.0222421.s002.pdf]

S1 ANALYSIS. CODING TREE

DATA REDUCTION

DESCRIPTIVE CODES		
Collaboration between health centres	Isolation of MDR-TB exposure	Isolation and patients' behaviour
Development since 2009	Psychiatry case	Knowledge of TB cases
Disclosure of HIV Status	Quantity of TB drugs	Lack of isolation facilities
DOTS' involvement	Size of drugs and adherence	Notification of TB
Family involvement	Suggestions for better treatment outcomes	OBs and Gynae case and clinical use
Fear of exposure of MDR-TB	Unavailability of TB and HIV medication	Patients attitude to health
Fear of exposure to TB infection	Unclear document and communication	Patients' socio-economic situation
Inadequate health education	Ward stock of TB drugs	Patients' personal TB/HIV medication
Insufficient staff	Disclosure of HIV status	Preparation for discharge
Interns' experience	Isolation and patient exposure	Private and public experience

DATA DISPLAY

INFERENTIAL CODES LEADING TO EMERGING THEMES

REVIEWER 1

CLINICAL MANAGEMENT OF TB

- **FEAR OF EXPOSURE TO TB INFECTION**
    - MDR-TB
        - Staff and Patients
            - Why?
    - Direct linkages
        - Clinical problems
            - Unclear knowledge of TB patients
                - Some lying in corridors
                - Not enough beds
            - Lack of proper isolation facilities
    - Indirect linkages
        - Patients' health-seeking behaviour
            - Inconsistent in-take (concealing drugs) of TB medication in hospital
            - Non-adherence/non-persistent to Treatment at home or at primary care
- **INADEQUATE HEALTH EDUCATION AND COMMUNICATION BETWEEN STAFF**
    - Unclear documentation of TB cases
        - Why?
    - Direct linkages
        - No standard procedure or not properly followed
        - Unclear allocation of tasks as to who should teach and when to teach
    - Indirect linkages
        - Insufficient time because of workload
        - Not enough staff
        - No electronic data-base of TB patients
- **UNIQUE EXPERIENCES IN PSYCHIATRY AND GYNAECOLOGY AND OBSTETRICS**
    - **Psychiatry-how?**
        - Proper coordination/team work between staff
        - The nurse takes the leading role in patient education

        - Clear documentation of patient information
        - Adequate contact with primary care using fax
        - Immediate follow-up of patients-linked to referrals category
        - **Why?** Not clear!!!!
    - **G & O – why?**
        - Very aware of the impact of TB on children
        - High-level of index because of reaching MDGs
        - Swift TB notification and dissemination of information

REFERRAL PROCESS

- **IMPROPER COLLABORATION BETWEEN HOSPITAL AND REFERRAL CLINICS**
    - Why?
- Direct linkages
    - Confusion on who should do more patient education
    - Inadequate knowledge of patient’s socio-economic situation affecting follow-up
    - No clear TB notification
    - No clear referral information
    - Inadequate transference of information to clinics as a result
- Indirect linkages
    - Patients’ understanding of TB information to take charge of continuing treatment
    - Language barrier-Translation of information when treating patients coming from other parts of Africa
    - Patients and family’s involvement in the process not clearly defined or adequate
- **SOCIO-ECONOMIC PROBLEMS OF PATIENTS**
    - How?
- Direct linkages
    - Poor living conditions
    - Lack of family support
    - inadequate understanding of TB message due to low educational level
- Indirect linkage
    - Poor health-seeking behaviour
    - Inadequate dissemination of patient housing location

SUGGESTIONS FOR BETTER TREATMENT OUTCOME

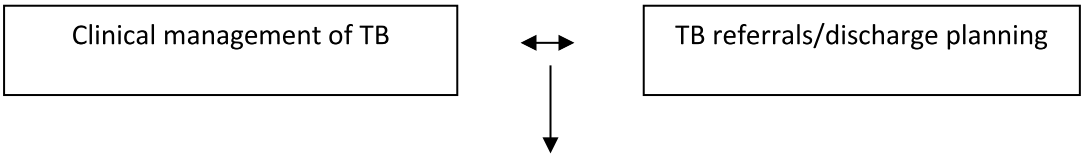

- Proper communication between staff treating TB patients-doctors and nurses
- Health care workers need to spend more time with patients-get to know their situation-be more empathetic
- Adequate communication between hospital and day clinic
- Have a TB coordinator/counsellor to coordinate TB referrals and link between the hospital and clinics, organises
- Standardise TB education/ information in a form of checklist/template
- Electronic data-base storing patient details

DATA DISPLAY

INFERENTIAL CODES LEADING TO EMERGING THEMES

REVIEWER 2

CARE MANAGEMENT PROCESS
FEAR OF EXPOSURE TO TB INFECTION

- **MDR-TB**
  - Staff and patients – occupational and environmental exposures
- **Clinical challenges**
  - Unclear/incomplete clinical/patient records
  - Lack of beds and respiratory isolation facilities
  - Low index of TB suspicion
  - Poor role modelling
- **Patient health-seeking behaviour**
  - Concealment of TB diagnosis/treatment
  - Poor treatment and isolation adherence in hospital
  - Poor treatment adherence at home or PHC

INADEQUATE TB EDUCATION AND SUPPORT FOR PATIENTS

- **Task shifting and not sharing**
  - Unclear understanding of roles, responsibilities and requirements
  - No standard approach for TB "induction" of patients
- **Insufficient time**
  - Workload
  - Inadequate number of staff (and with TB expertise)
- **Lack of health promoting resources**
  - Printed material and DVDs
  - Language and health literacy barriers

INADEQUATE TB COMMUNICATION BETWEEN STAFF

- **Poor documentation and coordination**
  - Absence/unclear documentation of TB cases (suspect/confirmed)
  - Partial handover on patient admission/transfer
  - Poor notification of TB cases
  - No electronic hospital database of all patients with TB
- **Hierarchy and territory**
  - Perceived professional parameters
  - Reluctance to track electronic results
  - Averseness to consult/confront the "other"

REFERRALS/DISCHARGE PROCESS
POOR DISCHARGE PLANNING

- **Lack of person-centredness and preparedness**
  - Inadequate assessment/knowledge of socio-economic conditions
  - Scanty patient/family/carer involvement in planning
  - Limited multi-disciplinary team consultation
  - Lack of linguistic and health literacy considerations
  - Deficient discharge information/instructions to patients/carers
- **Insufficient collaboration between hospital and referral points**
  - PHC level not included in discharge planning process
  - Unclear and/or incomplete discharge/referral information
  - Void of feedback on patients accessing PHC following discharge

SOCIO-ECONOMIC CONDITIONS OF PATIENTS

- **Poor living conditions**
  - Poverty
  - Conflicting survival priorities
  - Lack of family/carer support
  - Perpetual TB exposure (returning to "pool of infection")
- **Health literacy and agency**
  - Low education level
  - Poor health seeking behaviour
  - Submissive recipients of care
- **Strengthen TB-IPC**
  - Role modelling with accountability
  - Designated respiratory isolation facilities/wards
  - Rapid TB notification and contact screening
  - Sustain high index of suspicion ("TB in all")
- **Improved communication and coordination at and across hospital and PHC levels**
  - Role and responsibility clarification
  - Enhanced standard of documentation and verbal communication
  - Standardised TB documentation and procedures
  - TB Discharge Checklist/template
  - Electronic TB database
  - TB Coordinator/counsellor to manage person-centred CoC
  - Early involvement of/notification to PHC referral point
- **Improved TB education for patients and staff**
  - Integrate time in nursing care plan for patient health education
  - Health promoting resources for patients/families/carers
  - Sustained in-service training of broader aspects of TB care – for multidisciplinary healthcare staff (day, night and agency workers)
- **Person-centred discharge planning**
  - Assessment of socio-economic conditions (barriers and enablers)
  - Assessment of and respond to linguistic and health literacy requirements
  - Early engagement of patient and family/carer
  - Promote patient agency

VERIFYING CONCLUSIONS AND KEY THEMES BY BOTH REVIEWERS

CATEGORIES	KEY THEMES	SUB-THEMES
**Care Management Process**	Fear of exposure to TB infection	MDR-TB (Multidrug-Resistant TB)
		Clinical challenges
		Patient health-seeking behaviour
	Task shifting and not sharing	Insufficient time and staff shortage
		Lack of health education resources
	Poor documentation and coordination	
	Hierarchy and territory	
**Discharge and referral process**	Poor discharge planning	Lack of patient-centeredness and preparedness
		Insufficient collaboration between hospital and referral points
	Socio-economic conditions of the patients	Poor living conditions
		Health literacy and personal agency
**Participant recommendations for health system actions**	Strengthen TB-IPC	
	Improved communication at and across hospitals and PHC levels	
	Improved TB education for patients and staff	
	Patient-centered discharge planning	
